# Treatment of Detachment of the Retina

**Published:** 1897-02

**Authors:** 


### TREATMENT OF DETACHMENT OF THE RETINA.

*Casey A. Wood, in the Journal of the American Medical Association* of October 3, 1896, speaking of treatment in this condition, says detachment of the retina does not occur often in this country, but he has on his records six cases that consented to be treated for a sufficient length of time to make his experience with then worth mentioning. The treatment for two was scleral punctures, for one iridectomy, and the other three had prescribed for them continued rest in bed with pilocarpine injection. Only in one case, treated by puncture and pilocarpine, was there a field expanded, and the central vision improved to finger-counting at seven feet, although at one time it was reduced to perception of light. This improvement continued for nearly a year, when the patient was lost sight of.

Wood's experience leads him to think, with Bull of New York, that we have as yet discovered no better device than that resorted to with occasional success by the older ophthalmologists, viz.: rest in bed, bandages, atropine, and the internal use of some absorbent. Instead of the long-continued use of pilocarpine, especially when that drug is ill-borne by the patient, we may substitute soda bicarbonate and potassic iodide, well diluted with water. In all recent cases where the eye is quiet and there is no vitreous strand to sever, conjunctival puncture of the sclera may do temporary good and vision be improved. Division of fixed membranous bands in the vitreous may be done without causing much reaction, and may prevent extension of the disease. He does not approve Scholer's method.

Many cases of spontaneous cure are recorded; indeed, one may safely say that of all the histories of cures, temporary and permanent, at least ten per cent. were accomplished without treatment. So numerous and well authenticated are these recoveries that a large percentage of the results obtained after iridectomy, removal of the lens, the use of atropine, bandaging, pilocarpine, etc., and even in some cases, after posterior operation, are really brought about by local rest—by putting the patients in such a position that they can not, by overexertion of any kind, make a bad matter worse. The retina, having meantime broken loose from its connection with the shrinking vitreous, returns to its natural position—and the treatment, medical or surgical, receives the credit.—*Therapeutic Gazette.*

